# Identification of a New Major Oil Content QTL Overlapped with *FAD2B* in Cultivated Peanut (*Arachis hypogaea* L.)

**DOI:** 10.3390/plants14040615

**Published:** 2025-02-18

**Authors:** Feifei Wang, Huarong Miao, Shengzhong Zhang, Xiaohui Hu, Chunjuan Li, Weiqiang Yang, Jing Chen

**Affiliations:** Shandong Academy of Agricultural Sciences, Jinan 250100, China; wangfeifeisj@163.com (F.W.); huarm1969@163.com (H.M.); re_hunter@126.com (S.Z.); xiaohui800516@163.com (X.H.); peanutlab@163.com (C.L.); qdywq@126.com (W.Y.)

**Keywords:** peanut, oil content, QTL, *FAD2B*, candidate gene

## Abstract

High oil content in peanut seeds is a key breeding objective for peanut (*Arachis hypogaea* L.) quality improvement. In order to explore the genetic basis of oil content in peanuts, a recombinant inbred line (RIL) population consisting of 256 lines was phenotyped across six environments. Continuous distribution and transgressive segregation for both oil content and oleic acid content were demonstrated across all environments. Quantitative trait locus (QTL) analysis yielded 15 additive QTLs explaining 4.34 to 23.10% of phenotypic variations. A novel stable and major QTL region conditioning oil content (*qOCB09.1*) was mapped to chromosome B09, spanning a 1.99 Mb genomic region with 153 putative genes, including the oleic acid gene *FAD2B*, which may influence the oil content. Candidate genes were identified and diagnostic markers for this region were developed for further investigation. Additionally, 18 pairs of epistatic interactions involving 35 loci were identified to affect the oil content, explaining 1.25 to 1.84% of phenotypic variations. These findings provide valuable insights for further map-based cloning of favorable alleles for oil content in peanuts.

## 1. Introduction

Peanut (*Arachis hypogaea* L.) is widely cultivated worldwide as a food and oil crop. Peanuts are not only used as a rich source of vegetable oil, but are also rich in protein and micronutrients [1,2]. China is the world’s largest peanut producer, contributing to 34% of global production [3]; however, this output falls short of meeting the growing domestic demand for edible peanut oil [4]. Globally, approximately 55% of peanut production is used for oil extraction, while the rest is consumed as raw or processed food [5]. Significantly, a mere 1% rise in the oil content of peanuts can result in a considerable 7% economic enhancement for farmers and oil processing facilities alike [6]. Thus, high-oil-content peanut has become one of the key breeding objectives in China and other countries.

The oil content of peanut is a polygenetic inherited trait and is largely influenced by environmental factors such as temperature, light, water, and nutrition [7]. Unveiling the genetic basis of oil content is crucial for applying molecular markers in breeding programs to develop high-oil varieties. The significant variation (32.35–60.26%) in oil content among peanut germplasm means that there is a great potential to breed high-oil-content peanut varieties and provides an opportunity to identify the QTL/gene for oil content [8,9]. The peanut genome sequences were decoded in 2016 [10], while QTL mapping for peanut oil content began in 2009 [11]. The early mapping QTLs were based on genetic maps of SSRs utilizing RILs [6,12,13,14,15,16]. Although some KASP markers for both foliar fungal disease resistance and high oleic acid content have been developed and utilized in MAS [17], few markers were applied to oil content.

Thanks to the rapid development of sequencing technology, ddRAD-seq, whole-genome resequencing (WGRS), and specific-locus amplified fragment sequencing (SLAF-seq) have been applied to the detection of abundant SNPs for oil content QTL mapping [18,19,20,21]. Multi-omics, such as broad-target metabolomics, quantitative lipidomics, and transcriptome, have been employed to uncover the molecular mechanisms behind oil content [22,23]. Meanwhile, the reference genomes of diploid progenitors and cultivated peanuts were released [10,24,25,26], making it possible to construct high-density genetic maps. There are vast numbers of single nucleotide polymorphisms (SNPs) and insertions/deletions (InDels) that are widely distributed throughout the peanut genome. Several high-density genetic maps have been applied for peanut oil content QTL mapping [5,18,19,20,27,28]. To date, 36 major QTLs (PVE > 10%) for oil content have been identified [29], 8 of which are stable and can be detected across at least three environments on chromosomes A03, A05, and A08 [5,19,27].

Oleic acid (C18:1), one of eight fatty acids of oil, is an important oil quality trait for peanut. High oleic acid can improve the shelf life and health benefits (lower cholesterol in blood) for manufacturers and consumers, respectively [13,30]. High oil content and oleic acid content are both desirable peanut quality traits for peanut breeding. The *FAD2* genes, responsible for regulating oleic acid and the ratio between oleic acid and linoleic acid [31,32], may have no contribution to oil content [13,33].

In this study, the main objectives were to: (1) uncover stable QTLs for oil content across multiple environments and delimitate their physical regions in reference genomes, (2) estimate the contribution of *AhFAD2B* to oil content, (3) determine the effects from additive and epistatic loci and QTL × environment interaction, and (4) predict the candidate gene in the consistent QTL regions. This study will be valuable for elaborating the molecular mechanisms for oil content, which could be used to facilitate the breeding of high-oil-content peanut cultivars.

## 2. Results

### 2.1. Phenotypic Analysis of Kernel Oil and Oleic Acid Content in Parents and RIL Individuals

**Figure 1:**
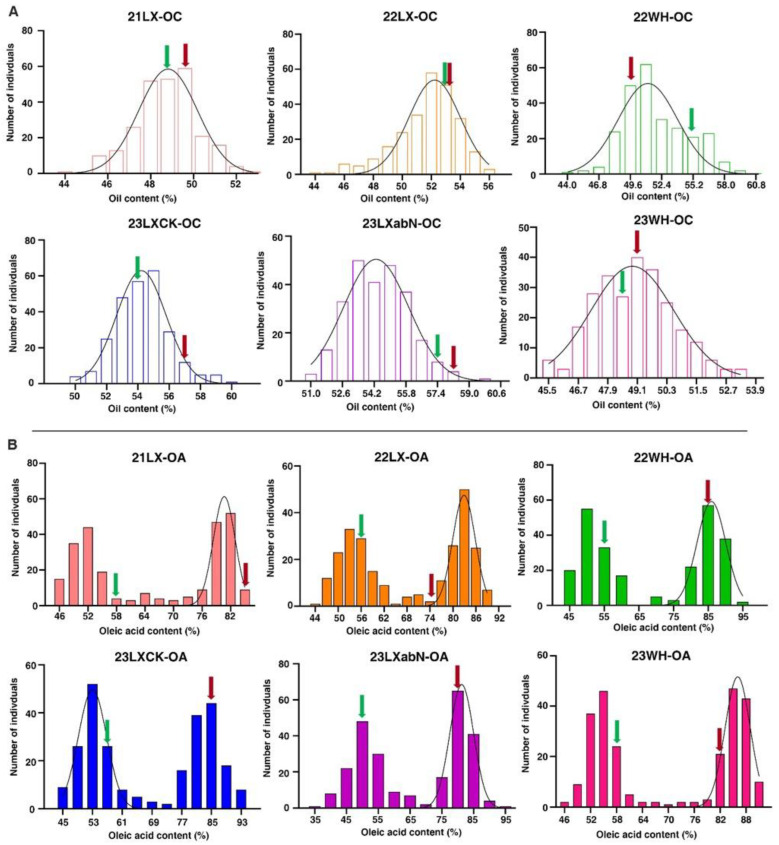
Phenotypic distribution of OC (**A**) and OA (**B**) in the RIL population across six environments. The y-axes represent the number of lines and the x-axes represent the values of oil content (OC) and oleic acid content (OA). 21LX, 22LX, 22WH, 23LXCK, 23LXabN, and 23WH were six trials in Laixi (LX) and Weihai (WH) from 2021 to 2023. Green and red arrows denote the parents, Luhua11 and 06B16, respectively.

Kernel oil and oleic acid content were assessed in 256 recombinant inbred lines (RILs) and their parental lines across six distinct environments: 2021LX, 2022LX, 2022WH, 2023LXCK, 2023LXabN, and 2023WH (Table 1). The results of their phenotypic values are displayed in Table 1. Although there was no difference between two parents for oil content in six different environments, significant differences were observed among the RILs for both traits across all six environments. The oleic acid content of 06B16 was significantly higher than that of Luhua11. The oil content in the RILs ranged from 43.52 to 60.10%, while the oleic acid content in the RILs ranged from 36.55 to 94.47%. The coefficient of variation (CV) of oil content ranged from 2.85 to 5.55%, while the CV of the oleic acid content in the RILs ranged from 1.35 to 1.56%. Remarkably, several RILs significantly surpassed their parents, Luhua11 and 06B16, in oil content and oleic acid, respectively (Figure 1).

The skewness and kurtosis of distribution are listed in Table 1, while the frequency distributions of individual phenotypic data for the RIL population and parents are displayed in Figure 1. Phenotypic values of both traits were found to have continuous distributions, and transgressive segregation occurred in the RIL population across all six environments. In each of the six environments, the population skewness and kurtosis (absolute value) in oil content were less than one, while kurtosis was more than one in oleic acid content. This result indicated that the segregations of oil content conformed to a skew normal or normal distribution model, with the characteristics of quantitative trait inheritance controlled by multiple genes. Furthermore, continuous distribution and considerable transgressive segregation observed in the RIL population demonstrated that both parents contributed alleles toward the oil and oleic acid content. All characteristics in the RIL population were confirmed to meet the requirements for QTL mapping.

The ANOVA (analysis of variance) results indicated that genotype (G), environment (E), and genotype by environment interaction (G × E) had significant effects on the oil and oleic acid contents (*p* < 0.01) (Table 2). The heritability of oil and oleic acid contents were 77% and 98%, respectively (Table 2).

### 2.2. Identification of QTLs for Oil Content and Oleic Acid Content

ICIM mapping identified a total of 26 additive QTLs associated with oil content (OC) and oleic acid content (OA) (Table 3). These 26 QTLs overlapped and were categorized into 16 loci. These loci were unevenly distributed on chromosomes A02 (1), A04 (2), A06 (1), A07 (3), B02 (2), B03 (3), and B09 (3), with LOD values of 2.50–80.34, and PVE ranges from 4.43 to 76.43% (Table 3).

**Figure 2:**
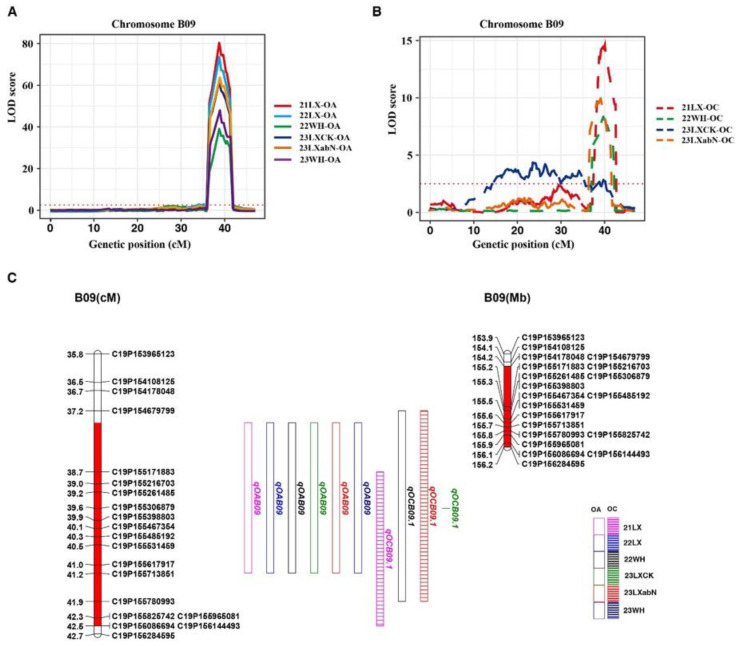
Colocalized QTL distribution of oil content (OC) and oleic acid (OA) on the genetic map. LOD curves of oleic acid content (**A**) and oil content (**B**) on the B09 chromosome across six environments. (**C**) Distribution of QTL for oleic acid content and oil content in the genetic map. The different colored boxes represent different environments. The stable and major QTL region is highlighted in red.

Oleic acid content in the RIL population was influenced by only one genomic region on chromosome B09, as shown in Table 3 and Figure 2. The phenotypic variance explained (PVE) by these QTLs ranged from 51.49 to 76.43%, with LODs of 39.59–80.34 in six environments (Table 3). The major stable QTL *qOAB09* showed negative additive effects, which were contributed by the paternal parent (06B16) allele, which exhibited high oleic acid content (Table 3).

A total of 15 QTL loci controlling oil content, namely *qOCA02*, *qOCA04.1*, *qOCA04.2*, *qOCA06*, *qOCA07.1*, *qOCA07.2*, *qOCA07.3*, *qOCB02.1*, *qOCB02.2*, *qOCB03.1*, *qOCB03.2*, *qOCB03.3*, *qOCB09.1*, *qOCB09.2*, and *qOCB09.3*, were detected on seven chromosomes (A02, A04, A06, A07, B02, B03, and B09) (Table 3). Among them, the major QTL *qOCB09.1* was repeatedly detected in 21LX, 22WH, 23LX-abN, and 23LX-CK, with the PVE ranging from 4.55 to 23.10%. The minor QTL *qOCA04.1* with 5.11% PVE was stably expressed in 23LX-abN and 23LX-CK. The remaining 14 QTLs were only detected in a single environment (Table 3). Except for *qOCB09.1*–*qOCB09.3*, all other QTLs showed negative additive effects, which were contributed by the male parent (06B16) allele (Table 3).

**Figure 3:**
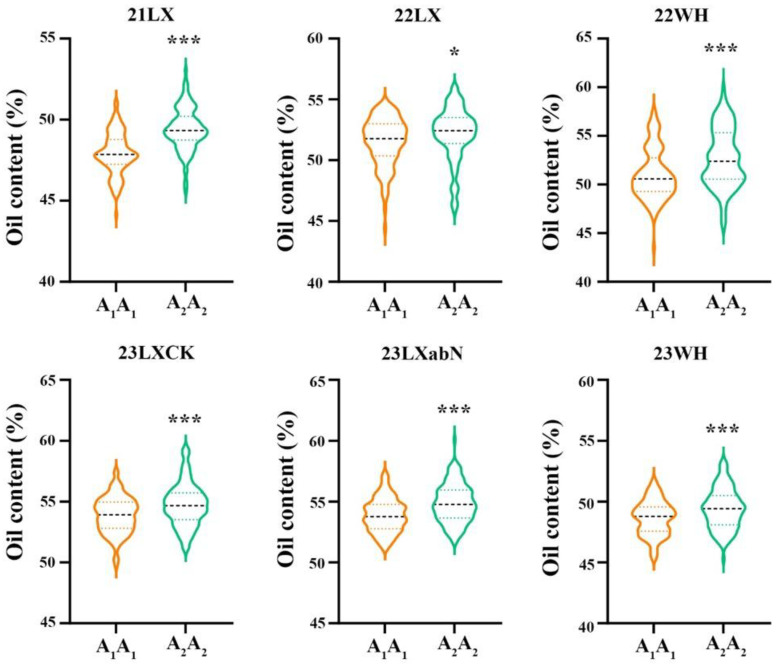
The violin plots for the oil content of two genotypic alleles for *qOCB09.1* across six different environments. A_1_A_1_ (yellow ochre violin) and A_2_A_2_ (green violin) represent the homozygous alleles in *qOCB09.1* from 06B16 and Luhua11, respectively. * and *** represent significant difference at *p* = 0.05 and 0.001 (Student’s *t*-test), respectively. 21LX, 22LX, 22WH, 23LXCK, 23LXabN, and 23WH were six trials in Laixi (LX) and Weihai (WH) from 2021 to 2023. The three lines in the violin plot represent the third quartile, median, and the first quartile, from top to bottom, respectively.

The confidence region of *qOCB09.1* was found in the mapped interval of 37.16–42.51 cM, which partially overlapped with *qOAB09* (Figure 2 and Table 3). According to the flanking markers of the detected QTLs, their physical intervals were determined using the reference genome of Tifrunner 2.0. We found that *qOCB09.1* and *qOAB09* were mapped in lower recombination regions on chromosome 19, and their physical interval was 155.16–156.19 Mb. To determine the phenotypic contributions of this major QTL region, the flanking marker profile was used to select and group RILs into two homozygous genotypes (Figure 3 and Table S3). A Student’s *t*-test revealed significant differences (*p* < 0.01) between the two genotypic groups across six environments (Figure 3). The RILs with the A_2_A_2_ genotype (representing the ‘Lu11’ allele in *qOCB09.1*) had a higher oil content than those with the A_1_A_1_ genotype (representing the ‘06B16’ allele in *qOCB09.1*). The oleic acid content of the two genotypes had the opposite results (Supplementary Figure S1).

### 2.3. The Recombination Effect of qOCB09 and qOCA04 for Oil Content

**Figure 4:**
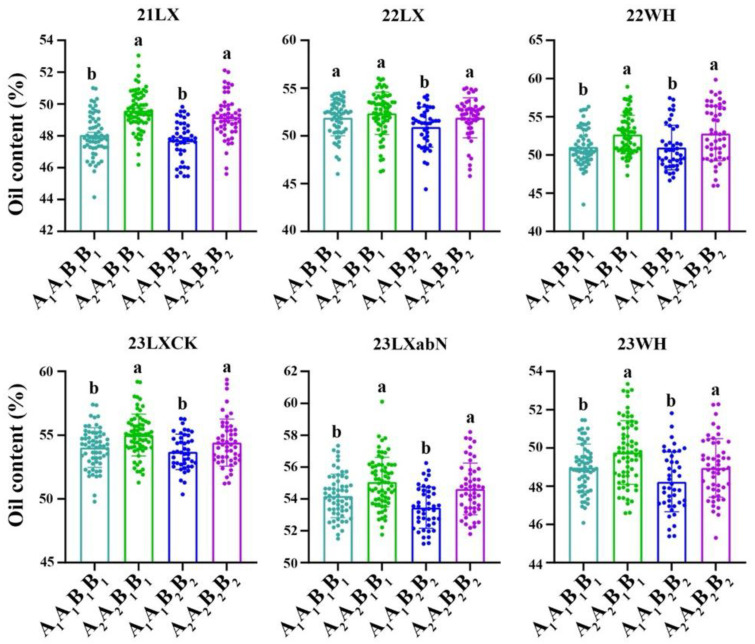
The combinatorial effects of QTLs on A04 and B09 across six environments. A and B represent QTL regions on B09 and A04, respectively. A_1_A_1_ and A_2_A_2_ represent the homozygous alleles in *qOCB09* from 06B16 and Luhua11, respectively. Different letters above the bars indicate significant (*p* < 0.05) differences among different combinatorial genotypes based on a one-way ANOVA test.

The RILs with the A_2_A_2_ genotype (representing the ‘Lu11’ allele in *qOCB09.1*) had a higher oil content than those with the A_1_A_1_ genotype (representing the ‘06B16’ allele in *qOCB09.1*) (Figure 3). The elite allele of qOCA04 from the elite ‘06B16’ was defined as the ‘B_1_B_1_’ genotype, while the allele from ‘Lu11’ was defined as the ‘B_2_B_2_’ genotype. We noticed that some RILs exhibited higher oil contents compared with both parents. Therefore, we further examined the distribution of alleles in two QTLs, *qOCA04* and *qOCB09*. The genotypes of two QTLs were ‘A_1_A_1_B_1_B_1_’ for parent ‘06B16’ and ‘A_2_A_2_B_2_B_2_’ for parent ‘Lu11’. There were four different genotypes, ‘A_1_A_1_B_1_B_1_’, ‘A_1_A_1_B_2_B_2_’, ‘A_2_A_2_B_1_B_1_’, and ‘A_2_A_2_B_2_B_2_’, from the lines in the RIL population. As expected, the lines with the ‘A_2_A_2_B_1_B_1_’ and ‘A_2_A_2_B_2_B_2_’ genotype created by combining two elite alleles of *qOCA04* and *qOCB09.1* showed an over-dominant phenotype with 52.41% and 51.98% of oil content, respectively, significantly higher than that of the lines with other genotypes (Figure 4 and Table S4). These results show that the combination of two elite alleles of *qOCA04* and *qOCB09.1* produced an over-dominant phenotype with significantly increased oil content.

The two loci associated with oil content were present in four genotype combinations: ‘A_1_A_1_B_1_B_1_’, ‘A_1_A_1_B_2_B_2_’, ‘A_2_A_2_B_1_B_1_’, and ‘A_2_A_2_B_2_B_2_’. The oil contents across the six environments were 48.05 ± 1.34%, 51.90 ± 1.88%, 51.05 ± 2.42%, 54.03 ± 1.56%, 54.19 ± 1.36%, and 48.97 ± 1.24% for the ‘A_1_A_1_B_1_B_1_ genotype; 49.58 ± 1.23%, 52.36 ± 2.22%, 52.67 ± 2.53%, 55.01 ± 1.64%, 55.06 ± 1.55%, and 49.76 ± 1.66% for the ‘A_1_A_1_B_2_B_2_’ genotype; 47.71 ± 1.22%, 50.93 ± 2.10%, 50.97 ± 2.91%, 53.69 ± 1.38%, 53.46 ± 1.30%, and 48.23 ± 1.57% for the ‘A_2_A_2_B_1_B_1_’ genotype; and 49.16 ± 1.36%, 51.89 ± 2.10%, 52.80 ± 3.56%, 54.42 ± 1.85%, 54.63 ± 1.63%, and 48.95 ± 1.53% for the‘A_2_A_2_B_2_B_2_’ genotype (Figure 4 and Table S4). A multiple comparison test indicated that the ‘A_2_A_2_B_1_B_1_’ genotype showed the highest oil content compared with the three other genotype combinations (‘A_1_A_1_B_1_B_1_’, ‘A_2_A_2_B_1_B_1_’, and ‘A_2_A_2_B_2_B_2_’). In particular, the oil content of the ‘A_2_A_2_B_1_B_1_’ genotype was 1.32–1.87% higher than that of the ‘A_1_A_1_B_2_B_2_’ genotype in different environments. Thus, it can be concluded that pyramiding favorable alleles of *qOCA04* and *qOCB09.1* could improve the oil content in peanut.

### 2.4. Detection of Epistatic QTLs for Oil Content

**Figure 5:**
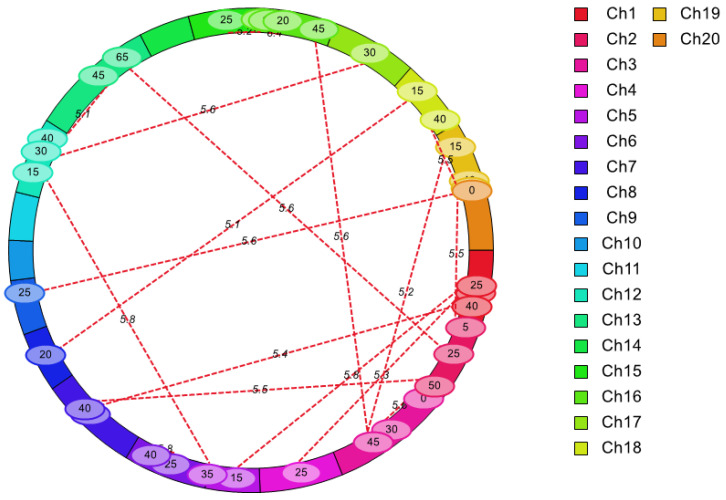
Epistatic QTL identified for oil content with the RIL population.

To further explore the epistatic effects of QTL on oil content, we analyzed the epistatic QTL with the ICIM-EPI mapping method using the MET module in IciMapping V4.1. A total of 18 pairs (35 loci) of epistatic QTLs regulating oil content were mapped on 17 LGs, with LOD values of 5.06–6.40 (Figure 5 and Table 4). The effect of additive-by-additive interaction varied from −0.25 to 0.20, and PVE of 1.25–1.84%. Only one epistatic locus on A03 could interact with two loci, whereas the other loci could only interact with a single locus.

### 2.5. Potential Candidate Genes for Oil Content in Major QTL Regions

**Figure 6:**
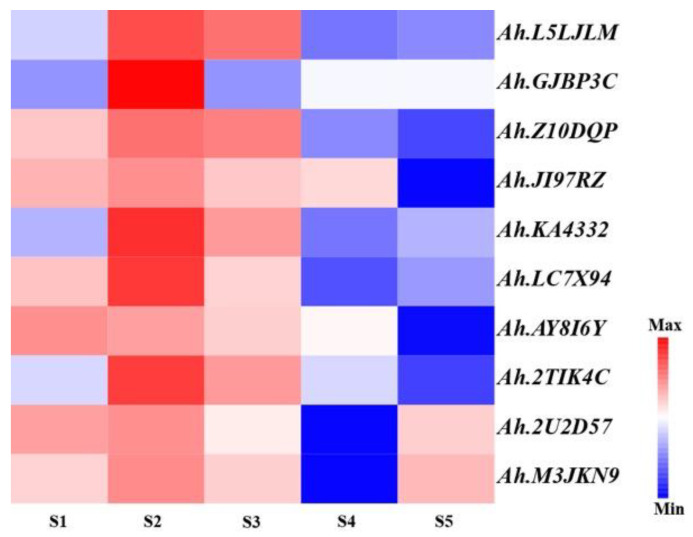
Heatmap analysis of candidate genes of peanut oil content in different developmental stages of kernel. S1 to S5 indicate the seed developmental stages 1 to 5. The values of transcript abundances were taken from [35]. The red and blue color indicate the upregulation and downregulation of candidate genes.

By retrieving gene models from the physical interval of the major QTL (PVE > 10%) for OC (Table 3), a total of 156 genes were obtained, 152 of which were annotated (Table S1). These genes were annotated with zinc finger protein, transcription factors, protein phosphatase, receptor-like protein kinase, zeaxanthin epoxidase, glycolipid transfer protein (GLTP) family protein, and fatty acid desaturase 2 (FAD2B) (Table S1). A total of 71 sequence variations (InDel or SNP) in the coding regions, intron, 5’UTR, or intergenic region were considered to be candidates for seed oil content (Table S2). Diagnostic markers for the candidate region were developed using PARMS technology, which demonstrated robust genotyping performance (Supplementary Figure S2). There were 15 genes with sequence variations in the genomic region of *qOCB09.1*/*qOAB09* including two transcription factor, three receptor-like protein kinase, transportin, Sec23/Sec24 protein transport family protein, putative nuclease HARBI1-like, zeaxanthin epoxidase, glycolipid transfer protein (GLTP) family protein, zinc finger protein CONSTANS-LIKE 16-like [34], acetyl glucosaminyl transferase family protein, alpha/beta fold hydrolase, and amino acid permease (Table S2). The expressions of all the candidate genes were highest at stage 2 of seed development, and decreased during stages 4 or 5 (Figure 6).

## 3. Discussion

### 3.1. QTL for Oil Content Was Stably Identified on Chromosome B09

In the past decade, with the rapid development of sequencing technology, a large number of studies have been carried out to map the oil content in peanut. Many QTLs for peanut oil content have been identified on all chromosomes including 36 major stable QTLs (PVE > 10%) on A03, A05, and A08. The hotspot QTL region is a 33.59–50.24 Mb genetic interval region on A08 [6,13,14,15,16,19,27,29]. The *qOCB02.1* in our study was located at 3.46–4.86 Mb on chromosome B02, consistent with *qAh12.1* at 3.5–3.6 Mb in Arahy12 [28]. *qOCB02.2* was located at 5.40–7.15 Mb on B02, and overlapped with the previously reported oil-content QTL in Arahy12 (6.6–7.5 Mb) [27]. *qOCB03.3* (131.04–135.43 Mb) was close to the previously discovered *qOCB03.2* (122.8–124.7 Mb) [18].

In a previous study, the oil content QTL *qOC-B09-1* was found to be located at 44–49 cM on B09, while the qOle-B09 for oleic acid content was found to be located at 82–87 cM on B09 [6]. Although the oil content was predicted through near-infrared, *qOCB09.1* was repeatedly detected in four environments, confirming the reliability of this result. The QTL region of *qOCB09.1* (37.16–42.51 cM) in the present study overlapped with *FAD2B*, so *qOCB09.1* is a new major stable QTL for oil content. This QTL has a PVE ranging from 4.55 to 23.10% and additive effects spanning from 0.43 to 0.96. As a result, we developed three diagnostic PARMS markers based on the SNPs for *qOCB09.1* (Supplementary Figure S2), intended to enhance marker-assisted selection for breeding high-oil-content peanut cultivars.

### 3.2. The Effect of AhFAD2B to Oil Content and Other Quality Traits

**Figure 7:**
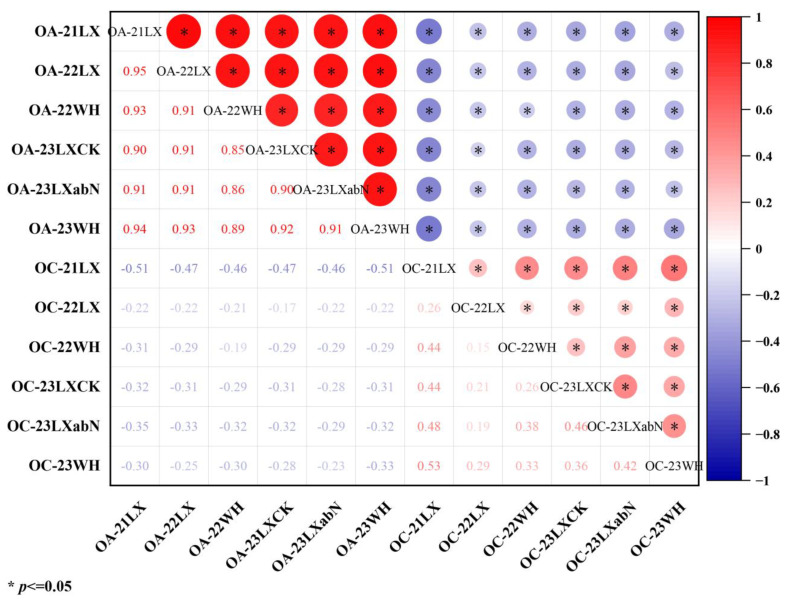
Correlation of oleic acid (OA) and oil content (OC) across six environments.

Unlike previous findings [15,28,33], the correlation analysis in this study showed a significantly negative correlation of OA with OC over all six environments (−0.19*–−0.51*) (Figure 7 and Table S5). *FAD2A/2B* are known for controlling oleic and linoleic acids, with *FAD2B* being a higher phenotypic contributor than *FAD2A* [13], and corresponding KASP markers have been developed [17]. There were no QTLs for oil content being colocalized with *FAD2*, with previous studies all showing that *FAD2* had no effects on the oil content [13,33]. In our study, we are the first to report that *qOCB09.1* for oil content and *qOAB09* for oleic acid content share the same QTL region. It is interesting that the *qSCB09*/*qSSCB09* for peanut seed sugar also overlapped with *qOCB09.1* [34], indicating that *FAD2B* may have an effect on the oil content and sugar content in peanut seed. A 205-bp MITE transpose element was inserted in the coding sequence of *FAD2B* (665bp) of the female parent ‘06B16’ [36]. The mobilization of AhMITE1 may be evoked by hybridization because de novo insertions in the hybrid progeny and mutant lines were detected [37]. Therefore, we hypothesized that the MITE transpose element in *FAD2B* of ‘06B16’ may be induced by hybridization and inserted to a gene around *FAD2B*, which regulates the oil content and sugar content. In the candidate physical interval of *qOCB09.1*, candidate gene *Ah.U2K3RD* encodes a dihydrolipoamide acetyltransferase component (E2) of pyruvate dehydrogenase complex that catalyzes pyruvate to acetyl coenzyme A, which is a direct precursor of fatty acid synthesis and may be the transformation hub of sugar, fat, and protein [38].

## 4. Materials and Methods

### 4.1. Plant Material and Growing Conditions

An F_2:6_ population of 256 RILs, derived from a cross of Luhua11 × 06B16, was used as a mapping population. The population was developed at the experimental station of Shandong Peanut Research Institute, Qingdao, China. The RIL population and its parental lines were planted in experimental fields in Laixi (N 36.86°, E 120.53°), Shandong Province (planted in May and harvested in September of 2021, 2022, and 2023) and Weihai (N 37.24°, E 122.37°), Shandong Province (planted in May and harvested in September of 2022 and 2023). Parental plants and 256 RILs were grown as previously described [34]. At maturity, seeds were harvested and air-dried from eight plants in the middle of each row.

### 4.2. Determination and Statistical Analysis of Phenotypic Data

About 20 seeds from each line and two parents were used to measure the oil and oleic acid concentration using near-infrared reflectance (NIR) spectroscopy, following the manufacturer’s protocol (Bruker Optics, Ettlingen, Baden-Württemberg, Germany). Statistical parameters for oil and oleic acid content among the RILs and two parents were separately calculated for each environment and combined environment using IBM^®^ SPSS^®^ Statistics 19 software (SPSS, Inc., Chicago, IL, USA). The normality of the population data was analyzed using the Kolmogorov–Smirnov test. Using the equation *h*^2^ = *σ*_g_^2^/(*σ*_g_^2^ + *σ*_ge_^2^/n + *σ*_ε_^2^/nr), the broad-sense of heritability (*h*^2^) for traits was calculated based on ANOVA analysis with QTL IciMapping V4.2 [39]. The frequency distribution graphs of phenotypic data were created using GraphPad Prism 9.0 (GraphPad Software, Inc., Boston, MA, USA).

### 4.3. QTL Mapping

A high-density genetic map was constructed for the mapping population, containing 3692 bin markers (82,292 SNPs) on 20 linkage groups (LGs) covering a total map distance of 981.65 cM, with an average marker distance of 0.27 cM [34]. QTLs were detected using composite interval mapping (CIM) through the R/qtl package [40]. The threshold of the logarithm of odds (LOD) value was determined by 1000 permutation tests at *p* = 0.05. MapChart 2.3 software was used to draw the genetic linkage map and QTLs [41]. The positive and negative additive effect that represented the favorable alleles were from Luhua11 and 06B16, respectively.

Epistatic interactions were detected using inclusive composite interval mapping of epistatic QTLs (ICIM-EPI) in the MET module through QTL IciMapping V4.1 software [39]. The LOD score was set by 1000 permutation tests, and specific parameters were set as: step = 5 cM, PIN = 0.001. The epistatic QTL was named as eq + the abbreviated name of a trait (OC) + LG number + ordered number on the LG [42].

### 4.4. Diagnostic Marker Development

In the stable and major QTL regions, SNPs and InDels were analyzed using peanut resequencing data. Markers displaying polymorphisms between the RIL parents were identified (Table S2). Three SNPs were employed to develop diagnostic markers by utilizing penta-primer amplification refractory mutation system (PARMS) technology. DNA extraction and PARMS genotyping of RIL samples was carried out as described by [43]. Primers used for PARMS are displayed in Supplementary Table S6.

### 4.5. Candidate Genes Mining

All of the genes in the QTL region were annotated in four databases (NR, Swiss-Prot, GO, KEGG). Gene prediction was according to the information on gene annotations obtained from Peanutbase (Arachis hypogaea cv. Tifrunner v1; https://www.peanutbase.org/, accessed on 8 July 2024). The relative expression levels of candidate genes in various tissues were obtained from previous studies [35].

## 5. Conclusions

In summary, we successfully identified a new major stable QTL region, qOCB09.1, for oil content on chromosome B09, explaining a phenotypic variance of 4.55% to 23.10%, and developed diagnostic PARMS markers. Surprisingly, qOCB09.1 overlapped with FAD2B, indicating that FAD2B may have an effect on oil content. Within this confidence interval, three non-synonymous mutation genes were identified as candidate genes. Epistasis analysis identified 35 genetic loci involving non-allelic interaction and controlling the oil variation. These findings contribute to an enhanced understanding of oil content in peanut, and the candidate genes will be useful for the breeding of high-oil-content peanut varieties.

## Figures and Tables

**Table 1:** Phenotypic variation of oil content (OC) and oleic acid (OA) among the RIL peanut populations in six environments.

Trait	Environment	Parents		RIL Population						
		Luhua11	06B16	Mean ± SD ^a^	Min ^b^	Max ^c^	CV ^d^ (%)	Skew	Kurt	Sig. of K-S Test ^e^
OC (%)	2021LX	48.70	49.37	48.72 ± 0.09	44.14	53.04	3.00	−0.102	0.087	0.200
	2022LX	53.48	53.54	51.71 ± 0.14	44.41	56.01	4.18	−0.792	0.604	0.000 **
	2022WH	55.86	49.50	51.86 ± 0.18	43.52	59.84	5.55	0.399	−0.362	0.000 **
	2023LXCK	53.73	57.07	54.32 ± 0.11	49.78	59.98	3.20	0.375	0.550	0.093
	2023LXabN	57.77	58.19	54.41 ± 0.10	51.19	60.10	2.85	0.343	−0.065	0.200
	2023WH	48.81	48.94	49.00 ± 0.10	45.30	53.34	3.29	0.211	−0.201	0.200
	Mean ± SD	53.06 ± 3.68	52.77 ± 4.13							
OA (%)	2021LX	57.52	84.94	65.91 ± 0.91	45.16	85.68	1.38	−0.033	−1.815	0.000 **
	2022LX	55.47	75.18	68.21 ± 0.92	44.53	89.68	1.35	−0.046	−1.733	0.000 **
	2022WH	53.69	83.38	68.55 ± 1.07	43.05	94.47	1.56	0.001	−1.773	0.000 **
	2023LXCK	54.93	83. 44	68.61 ± 0.98	43.01	93.90	1.43	−0.022	−1.708	0.000 **
	2023LXabN	50.87	78.87	66.65 ± 0.99	36.55	92.88	1.49	−0.138	−1.665	0.000 **
	2023WH	58.50	82.86	70.15 ± 0.99	46.82	92.30	1.41	−0.011	−1.862	0.000 **
	Mean ± SD	55.16 ± 2.74	80.45 ± 3.68 **							

^a^ SD, standard deviation; ^b^ Min, minimum value; ^c^ Max, maximum value; ^d^ CV, coefficient of variation; ^e^ Sig of K–S test, significance for normality test by Kolmogorov–Smirnov; ** means significant at *p* < 0.01. 2021LX, 2022LX, 2022WH, 2023LXCK, 2023LXabN, and 2023WH were six trials in Laixi (LX) and Weihai (WH) from 2021 to 2023; 2023LXabN denotes the absence of N fertilization in 2023LX.

**Table 2:** Analysis of broad-sense heritability of oil content (OC) and oleic acid (OA) of peanut.

Traits	Source	DF ^a^	SS ^b^	MS ^c^	F Value	*p*	h^2^
OC	G	255	4672.90	18.33	30.52	<0.01	0.77
	E	5	12,607.47	2521.49	4199.00	<0.01	
	G × E	1268	6804.38	5.37	8.94	<0.01	
	Error	1521	913.36	0.60			
OA	G	255	676,577.13	2653.24	196.19	<0.01	0.98
	E	5	5994.31	1198.86	88.65	<0.01	
	G × E	1268	63,225.74	49.86	3.69	<0.01	
	Error	1521	20,569.35	13.52			

^a^ DF degree of freedom; ^b^ SS sum of square; ^c^ MS mean of square.

**Table 3:** Unconditional QTL analysis for oil content (OC) and oleic acid (OA) contents.

Trait	Env ^a^	QTL	LG ^b^	CI ^c^	Flanking Markers	Physical Position (Mb)	LOD ^d^	PVE ^e^	ADD ^f^ (%)
OC	23LX-CK	*qOCA02*	A02	12.92–13.34	C02P3759859–C02P3916184	3.62–3.93	2.81	4.94	0.35
	23LX-abN	*qOCA04.1*	A04	17.72–19.60	C04P5985371–C04P7532051	5.95–7.65	3.50	6.12	−0.34
	23LX-abN	*qOCA04.2*	A04	26.75–26.75	C04P89232398–C04P89232398	89.22–89.25	2.51	4.43	−0.29
	23LX-CK		A04	23.07–23.07	C04P10564647–C04P10564647	10.54–10.59	2.50	4.40	−0.24
	23LX-CK		A04	23.67–27.59	C04P12663865–C04P101171840	11.95–102.52	3.74	6.50	−0.35
	23LX-abN	*qOCA06*	A06	11.78–16.49	C06P4952448–C06P6221608	4.93–6.24	4.97	8.59	−0.43
	23LX-abN	*qOCA07.1*	A07	18.14–18.35	C07P3648882–C07P3718138	3.61–3.74	2.53	4.47	−0.25
	23LX-abN	*qOCA07.2*	A07	21.23–21.64	C07P4031007–C07P4125216	4.02–4.13	2.63	4.65	−0.27
	23LX-abN	*qOCA07.3*	A07	35.83–35.83	C07P12259068–C07P12259068	12.15–12.37	2.52	4.45	−0.29
	23LX-abN	*qOCB02.1*	B02	2.81–7.56	C12P3505471–C12P4779507	3.46–4.86	5.23	9.02	−0.46
	23LX-CK	*qOCB02.2*	B02	10.78–15.57	C12P5449276–C12P7073350	5.40–7.15	5.42	9.30	−0.47
	23LX-abN	*qOCB03.1*	B03	63.10–63.73	C13P136851060–C13P137089223	136.84–137.12	2.79	4.91	−0.36
	23LX-abN	*qOCB03.2*	B03	64.76–64.76	C13P137639416–C13P137639416	137.55–137.73	2.53	4.47	−0.35
	23LX-CK	*qOCB03.3*	B03	53.71–58.49	C13P131095813–C13P135374538	131.04–135.43	5.46	9.35	−0.53
	21LX	*qOCB09.1*	B09	38.74–42.51	C19P155171883–C19P156144493	155.16–156.19	14.60	23.10	0.70
	22WH		B09	37.16–41.85	C19P154679799–C19P155780993	154.20–155.80	7.72	13.12	0.96
	23LX-abN		B09	37.16–41.85	C19P154679799–C19P155780993	154.20–155.80	9.41	15.63	0.54
	23LX-CK		B09	39.63–39.63	C19P155306879–C19P155306879	155.27–155.34	2.59	4.55	0.43
	23LX-CK	*qOCB09.2*	B09	15.38–29.46	C19P11107052–C19P145410341	11.03–14.54	4.10	7.12	0.43
	23LX-CK	*qOCB09.3*	B09	30.35–35.18	C19P147560425–C19P153510614	147.54–153.61	3.39	5.92	0.44
OA	21LX	*qOAB09*	B09	36.50–41.18	C19P154108125–C19P155713851	154.06–155.76	80.34	76.43	−12.31
	22LX		B09	36.50–41.18	C19P154108125–C19P155713851	154.06–155.76	73.29	73.66	−12.19
	22WH		B09	36.50–41.18	C19P154108125–C19P155713851	154.06–155.76	39.59	51.49	−13.65
	23LX-CK		B09	36.50–41.18	C19P154108125–C19P155713851	154.06–155.76	61.43	66.88	−12.40
	23LX-abN		B09	36.50–41.18	C19P154108125–C19P155713851	154.06–155.76	65.96	69.62	−12.76
	23WH		B09	36.50–41.18	C19P154108125–C19P155713851	154.06–155.76	48.17	71.64	−11.66

^a^ Env environment; ^b^ LG linkage group; ^c^ Cl confidence interval; ^d^ LOD logarithm of the odds; ^e^ ADD additive effect; ^f^ PVE phenotypic variation explained.

**Table 4:** Analysis of interaction effects of epistatic QTL and environment on oil content (OC) across six environments.

Trait/QTL	Locus 1				Locus 2				LOD	PVE	Add1	Add2	AddbyAdd
	Chr1	Position1 (cM)	LeftMarker1	RightMarker1	Chr 2	Position2 (cM)	LeftMarker2	RightMarker2					
*eqOCA03-1*	A03	0	C03P758466	C03P1532569	A03	30	C03P30050409	C03P30278461	5.58	1.49	−0.02	0.00	0.20
*eqOCA01-1*	A01	30	C01P101103927	C01P101214504	A04	25	C04P30035679	C04P31759622	5.29	1.47	−0.16	0.24	−0.05
*eqOCA01-2*	A01	25	C01P98303028	C01P98374108	A05	15	C05P40841414	C05P41229144	5.78	1.65	−0.13	0.01	−0.09
*eqOCA06-1*	A06	25	C06P9796191	C06P10122726	A06	40	C06P98814906	C06P99212948	5.80	1.44	−0.02	−0.10	0.20
*eqOCA01-3*	A01	40	C01P105905716	C01P107392629	A07	35	C07P11276815	C07P11495248	5.38	1.33	−0.11	0.13	−0.19
*eqOCA02-1*	A02	50	C02P96741356	C02P96784311	A07	40	C07P41451767	C07P43171362	5.49	1.32	0.01	0.19	−0.14
*eqOCA05-1*	A05	35	C05P106246855	C05P108041290	B02	15	C12P6972241	C12P7073350	5.78	1.77	−0.01	0.06	−0.17
*eqOCB02-1*	B02	40	C12P111880113	C12P112384452	B03	45	C13P42640495	C13P42898862	5.06	1.26	−0.10	0.08	0.17
*eqOCA02-2*	A02	25	C02P9045260	C02P9433222	B03	65	C13P137756553	C13P137866024	5.61	1.31	0.05	0.10	−0.18
*eqOCB05-1*	B05	25	C15P151189954	C15P151265988	B06	5	C16P2025227	C16P2053025	5.16	1.84	0.01	0.00	0.13
*eqOCB06-1*	B06	10	C16P6973672	C16P7029871	B06	20	C16P9506414	C16P10163123	6.40	1.25	−0.04	0.05	−0.25
*eqOCA03-2*	A03	45	C03P128973442	C03P129309550	B06	45	C16P141581457	C16P142022545	5.55	1.36	−0.02	−0.02	−0.19
*eqOCB02-2*	B02	30	C12P97757157	C12P100511712	B07	30	C17P21849598	C17P21979066	5.64	1.64	−0.02	−0.07	0.15
*eqOCA08-1*	A08	20	C08P29541313	C08P29694890	B08	15	C18P7671897	C18P7868227	5.07	1.39	0.10	−0.04	−0.18
*eqOCA03-3*	A03	45	C03P128973442	C03P129309550	B09	15	C19P10437570	C19P11107052	5.16	1.37	0.00	−0.06	−0.19
*eqOCA02-3*	A02	5	C02P2187686	C02P2282492	B09	40	C19P155398803	C19P155467354	5.53	1.25	0.03	−0.26	0.18
*eqOCA09-1*	A09	25	C09P114246497	C09P114513794	B09	45	C19P156650243	C19P156732175	5.55	1.46	0.01	0.02	0.18
*eqOCB08-1*	B08	40	C18P129515227	C18P129730247	B10	0	C20P899663	C20P1879165	5.48	1.53	−0.08	0.00	−0.17

## Data Availability

Data will be made available on request.

## Supplementary Materials

The following supporting information can be downloaded at: https://www.mdpi.com/article/10.3390/plants14040615/s1, Figure S1: The oleic acid content of two genotypic alleles for qOAB09 across six different environments; Figure S2: Penta-primer amplification refractory mutation system (PARMS) genotyping results.; Table S1: Function annotation of genes in the major QTL region of OC; Table S2: The Indel and SNPs differ in two parents winthin major QTL regions of OC content and OA content; Table S3: The phenotype of Bin markers in three QTL regions for OC/OA; Table S4: Joint effect of two QTLs (*qOCB09.1* and *qOCA04*); Table S5: The correlation of kernel sugar content, oleic acid content, and oil content around six environments; Table S6: Primers used for PARMS and qRT-PCR analyses.
