# Supplementary material for: Identification of a New Major Oil Content QTL Overlapped with FAD2B in Cultivated Peanut (Arachis hypogaea L.)
Source: Plants (Basel). 2025 Feb 18;14(4):615. doi: 10.3390/plants14040615 (PMC11859173; doi:10.3390/plants14040615)
Supplement: Supplementary file 1 [file plants-14-00615-s001.zip › supplemental Figures.pdf]

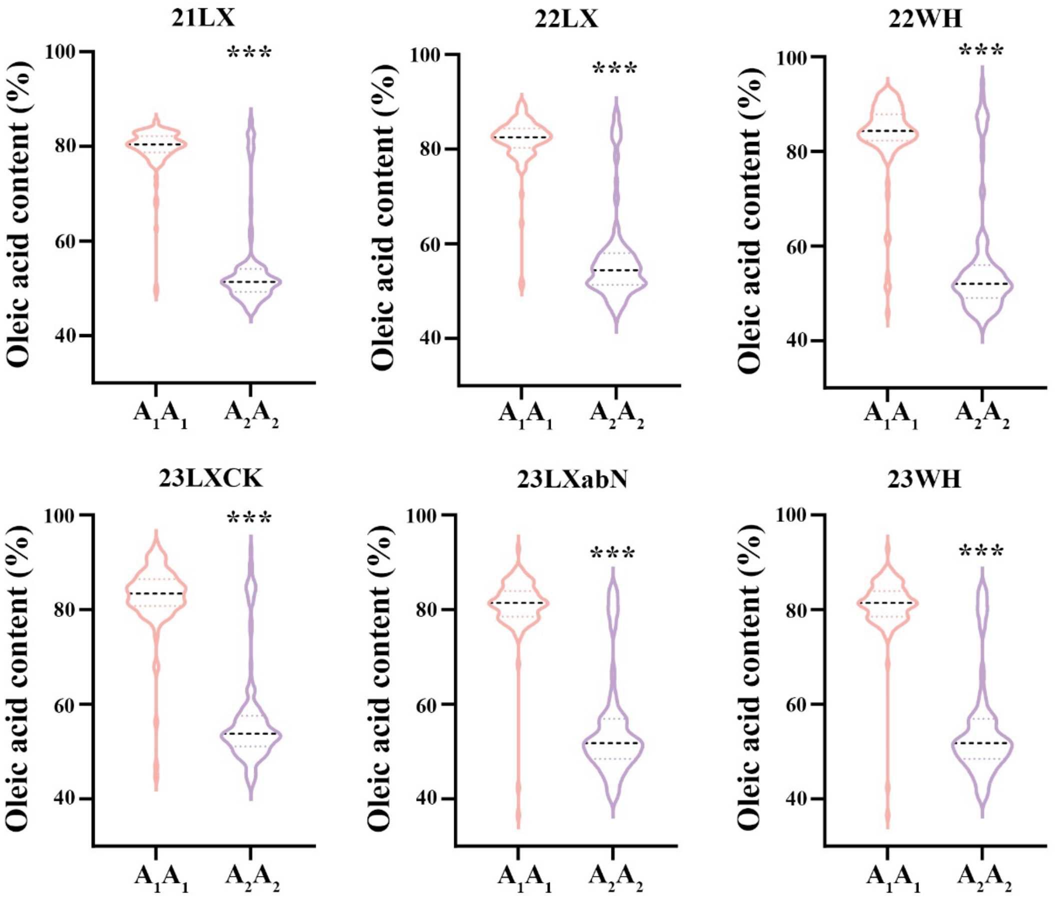

Supplementary Fig. S1 The oleic acid content of two genotypic alleles for *qOAB09* across six different environments. A_1A_1 and A_2A_2 represented the homozygous alleles in *qOAB09* were from 06B16 and Lu11, respectively. *** represent significant difference at P= 0.001 level by t-test.

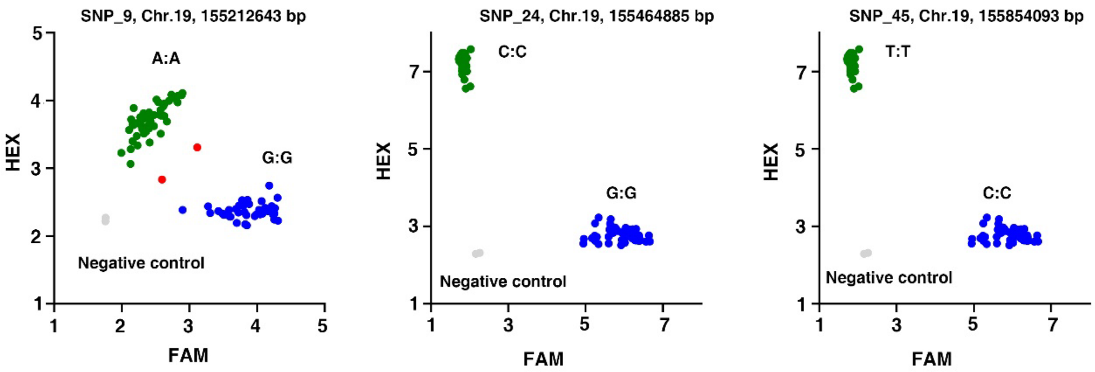

Supplementary Fig. S2 Penta-primer amplification refractory mutation system (PARMS) genotyping results. SNP_4, SNP_24 and SNP_45 belongs to the bin markers C19P155216703, C19P155467354 and C19P155965081, respectively. Green dot and blue dots indicate the genotypes of 'Luhua11' and '06B16', respectively.
